# Epitranscriptomic Role of m6A in Obesity-Associated Disorders and Cancer Metabolic Reprogramming

**DOI:** 10.3390/genes16050498

**Published:** 2025-04-27

**Authors:** Sujun Yan, Weijing Wen, Zhe Mo, Simeng Gu, Zhijian Chen

**Affiliations:** 1 Zhejiang Provincial Center for Disease Control and Prevention, 3399 Bin Sheng Road, Binjiang District, Hangzhou 310051, China; jsyan@cdc.zj.cn (S.Y.); wwwenjing27@outlook.com (W.W.); zhmo@cdc.zj.cn (Z.M.); smgu@cdc.zj.cn (S.G.); 2 School of Public Health, Health Science Center, Ningbo University, 818 Feng Hua Road, Jiangbei District, Ningbo 315211, China

**Keywords:** m^6^A, epigenetic, adipose tissue, metabolism, obesity, insulin resistance

## Abstract

The global rise in obesity and its associated metabolic disorders underscores the need for a deeper investigation into their underlying molecular mechanisms. While genetic factors are well-established contributors, recent research has increasingly focused on epigenetic regulators, particularly N6-methyladenosine (m^6^A)—the most prevalent internal RNA modification in eukaryotes. This post-transcriptional modification plays a crucial role in RNA metabolism by regulating mRNA stability, splicing, nuclear export, and translation efficiency. Notably, emerging evidence implicates m^6^A in both adipogenesis and metabolic dysregulation. In this review, we systematically examine three key dimensions: (1) the molecular mechanisms of m^6^A modification, including writers, erasers, and readers, in obesity; (2) dysregulated m^6^A patterns in obesity-related pathologies, such as type 2 diabetes (T2D), insulin resistance, metabolic dysfunction-associated steatotic liver disease (MASLD), and the glycolysis in cancer cells; and (3) the therapeutic potential of targeting m^6^A and the regulators. By critically assessing recent advancements, we highlight m^6^A’s dual role as both a metabolic sensor and a disease modulator, offering novel insights into potential strategies for combating obesity-related metabolic syndromes.

## 1. Introduction

Over the past few decades, obesity has emerged as a global public health crisis, profoundly affecting quality of life, increasing the risk of chronic diseases, and imposing a growing economic burden on healthcare systems worldwide [1,2,3]. Since 1975, the global prevalence of obesity has nearly tripled, with a striking shift in mortality patterns, obesity-related deaths now exceed those caused by undernutrition and underweight conditions [4]. Beyond its physical manifestations, obesity disrupts systemic organ function and serves as a major driver of life-threatening comorbidities, including diabetes, insulin resistance, hepatic steatosis, and cardiovascular diseases [5]. Population-based studies further highlight the strong association between obesity-related complications and increased mortality risk [6]. Given its widespread impact on human health and socioeconomic stability, developing effective strategies for obesity prevention and treatment is imperative to mitigate future health and economic consequences.

Obesity is a multifactorial disorder shaped by complex interactions between genetic, epigenetic, and environmental factors [6,7,8,9,10]. While environmental influences such as diet and lifestyle play undeniable roles, accumulating evidence underscores the strong heritability of common obesity. Genetic studies have identified key obesity-associated genes involved in appetite regulation, insulin secretion, adipogenesis, lipid metabolism, and pathways linked to obesity-related metabolic dysfunction [11,12,13]. Notably, specific epigenetic markers, especially those governing energy metabolism, demonstrate transgenerational inheritance, positioning them as potential determinants of obesity [14,15]. These insights highlight epigenetic regulation as a promising therapeutic frontier for obesity management.

Among epigenetic modifications, N6-methyladenosine (m^6^A) has garnered significant attention as the most prevalent and reversible post-transcriptional RNA modification [16]. The dynamic regulation of m^6^A involves three key protein classes: “writers”, which catalyze methylation; “erasers”, which remove methylation; and “readers”, which interpret m^6^A to modulate mRNA metabolism [17]. Emerging evidence implicates m^6^A in a wide range of biological processes, including tumorigenesis, inflammation, and metabolic disorders [3,18]. Interestingly, reduced m^6^A levels have been observed in obese tissues [15], suggesting a crucial role for m^6^A in obesity pathogenesis and metabolic dysregulation.

This review provides a comprehensive analysis of the role of m^6^A in obesity and its associated metabolic disorders in recent years, clarifying its mechanistic contributions and identifying future research directions to leverage this epigenetic pathway for therapeutic innovation.

## 2. Overview of m^6^A Methylation Modification

Over 150 RNA modifications have been identified as markers of various post-transcriptional processes in RNA [19,20]. These modifications, ranging from well-established to newly discovered, play critical roles in regulating RNA transcription and metabolism, including alternative splicing, RNA transport, stability, and translation processes [21,22,23,24]. Studies have shown that N6-methyladenosine (m^6^A) modification is the most abundant and dynamically regulated modification in the transcriptomes of eukaryotes, including yeast, plants, insects, and mammals [25,26,27,28]. In 1970, scientists first discovered the presence of m^6^A [29]. However, due to the lack of methods to detect m^6^A sites, the research on m^6^A has been stalled. In 2012, a breakthrough came with the advent of m^6^A-specific immunoprecipitation (m^6^A-IP) combined with high-throughput sequencing. This innovative approach revealed that m^6^A is a prevalent mRNA modification, with at least 25% of human transcripts containing over 10,000 m^6^A peaks [30,31]. m^6^A selectively marks regions near stop codons and 3′ untranslated regions (UTRs), following the RRACH consensus motif (R: A/G; H: A/U/C) [31]. Studies showed that m^6^A is dynamically regulated, varying with development and cellular stress [32,33], highlighting its critical roles in RNA metabolism and spurring renewed interest in its functions.

**Figure 1:**
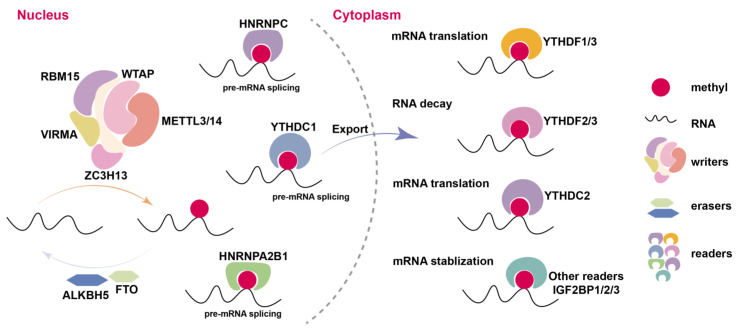
The dynamic process of m^6^A modification and its regulatory role.

The abundance and regulation of m^6^A on RNA are governed by the dynamic interplay between methyltransferases (“writers”), demethylases (“erasers”), and recognition proteins (“readers”) [17] (Figure 1). METTL3, the first identified m^6^A methyltransferase [17,34], functions in tandem with METTL14 and the adaptor protein WTAP to mediate m^6^A deposition [35,36,37,38]. METTL14 enhances the catalytic activity of METTL3 by acting as an RNA-binding scaffold, while WTAP is crucial for the proper localization of the METTL3–METTL14 complex to nuclear speckles [35,37]. KIAA1429 (VIRMA) plays a pivotal role in recruiting the METTL3–METTL14–WTAP complex to specific RNA regions, thereby modulating the deposition of m^6^A [39,40,41,42]. Additionally, RNA Binding Motif Protein 15 (RBM15) and RBM15B direct the methyltransferase complex to RRACH motifs by binding to guanine-rich regions [42,43]. Recent studies have identified that Zinc Finger CCCH-Type Containing 13 (ZC3H13) and HAKAI have further expanded our understanding of the composition and regulatory mechanisms governing the m^6^A methyltransferase complex [39,44]. The primary m^6^A demethylases are FTO and ALKBH5. FTO preferentially catalyzes the demethylation of m^6^Am (methylated N6-adenosine at the 5′ cap structure) [45,46], whereas ALKBH5 specifically targets m^6^A and plays a critical role in regulating mRNA stability and nuclear export [47]. The loss of FTO or ALKBH5 leads to a marked increase in global m^6^A levels or a reduction in the nuclear polyadenylated RNA pool, respectively [45,46,47]. Although m^6^A modification in RNA transcripts is regulated by the coordinated actions of methyltransferases and demethylases, it is the recognition by reader proteins that confers the diverse biological functions of m^6^A [19,48,49,50,51]. The YTH (YT521-B homology) domain-containing proteins are the primary m^6^A readers, responsible for mediating the post-transcriptional regulation of m^6^A-modified RNAs. The members of the DF family (YTHDF1, YTHDF2, and YTHDF3) share high sequence similarity and are predominantly located in the cytoplasm [51,52,53]. YTHDF1 promotes mRNA translation, with some studies suggesting its involvement in mRNA degradation under specific conditions [22,54,55]. YTHDF2, in contrast, primarily induces the destabilization of m^6^A-modified mRNAs, playing a central role in mRNA decay [54]. YTHDF3 acts to coordinate the functions of both YTHDF1 and YTHDF2, thus fine-tuning the balance between mRNA translation and degradation [55,56,57]. In the nucleus, YTHDC1 regulates mRNA splicing and export, ensuring proper processing and trafficking of mRNA [21,24,52,58]. YTHDC2, which enhances translation efficiency while reducing target mRNA abundance, has a more controversial role in m^6^A binding [59,60]. eIF3 contributes to translation by interacting with the 5′UTR or associating with YTHDF1 [22,55]. The role of HNRNPA2B1 as a potential m^6^A reader remains controversial, with conflicting evidence regarding its function in the m^6^A-regulatory process [61,62].

## 3. The Role of m^6^A in Regulating Obesity

Obesity primarily arises when energy intake exceeds energy expenditure, leading to the accumulation of excess energy in the form of triglycerides. Adipose tissue, once regarded as a passive energy reservoir, was redefined in the 1980s as a central metabolic organ [63]. With advancing research, accumulating evidence has demonstrated that adipose tissue development and metabolism are intricately involved in various biological processes, including metabolic regulation and body weight homeostasis [5]. As early as 2007, genome-wide association studies identified FTO as a gene associated with obesity [64,65]. However, the underlying mechanisms driving adipose tissue expansion and metabolism remained unclear. In 2011, FTO was discovered to possess demethylase activity for m^6^A modification. This finding spurred further investigations into the role of m^6^A-mediated regulation in obesity [16,45].

### 3.1. M^6^A Regulates Adipogenesis

Adipogenesis is a complex process that occurs in two key stages. The first stage involves the transformation of pluripotent stem cells into preadipocytes in response to specific signaling cues [12,66,67]. The second stage is characterized by terminal differentiation, mediated by a series of transcription factors and epigenomic regulators. When adipocyte growth is limited, the process activates key adipogenic regulators, including peroxisome proliferator-activated receptor γ (PPAR-γ) and the transcriptional coactivators CCAAT/enhancer-binding protein α (C/EBPα) and β (C/EBPβ), which promote lipid accumulation and cellular maturation [68].

Mesenchymal stem cells (MSCs) demonstrate robust self-renewal potential and can differentiate into adipocytes, chondrocytes, and osteoblasts. Emerging evidence has demonstrated m^6^A as a critical epigenetic modulator of this process. In vitro experiments have shown that deletion of METTL3 in porcine bone marrow stem cells (BMSCs) could promote adipogenesis [69]. The removal of *Mettl3* in BMSCs resulted in a significant increase in BMSC adipogenesis. This effect was attributed to the downregulation of AKT1 expression, an AKT serine/threonine kinase 1, in an m^6^A-dependent manner [70]. Similarly, WTAP promoted osteogenesis and inhibited adipogenesis of BMSCs via the WTAP/miR-29b-3p/HDAC4 pathways in an m^6^A-dependent manner [71]. Furthermore, FTO has been shown to demethylate m^6^A modification on *PPARγ* mRNA, thereby upregulating its expression and promoting the shift of osteoporotic BMSC fate to adipocytes [72]. Recent studies have also revealed that ALKBH5-mediated m^6^A modification increases the expression of *TRAF4* (TNF receptor-associated factor 4) mRNA, thereby promoting the adipogenic differentiation of MSCs [73]. IGF2BP3 interacted with *MYLK* mRNA in an m^6^A-dependent manner, extending its half-life and subsequently inhibiting the phosphorylation of the ERK1/2 pathway, thereby impeding the adipogenic differentiation of MSCs [74]. Collectively, these findings highlight the involvement of m^6^A in the regulation of adipogenesis in BMSCs.

**Figure 2:**
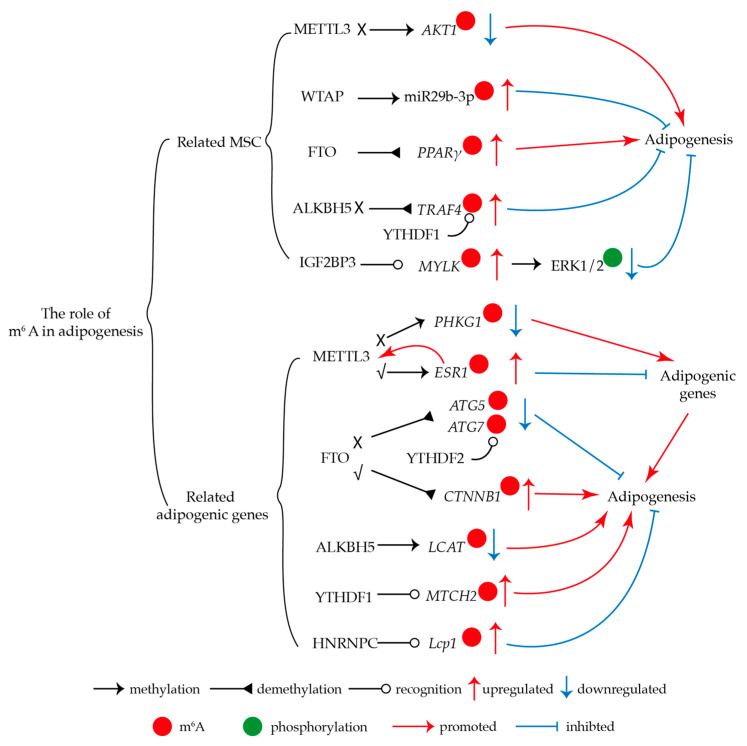
M^6^A regulates adipogenesis.

Studies have suggested that overexpressed METTL3 inhibits adipogenesis, relying on the increased m^6^A modification, indicating that m^6^A may be a critical regulator in this process [75]. This represents the first evidence linking m^6^A modification to the regulation of adipogenesis. The recent study also showed that knockdown of the methylase METTL3 decreased the m^6^A methylation of PHKG1 and led to a reduction in PHKG1 and then promoted adipogenic differentiation by upregulating the expression of adipogenic genes [76]. METTL3-mediated m^6^A modification stabilized Estrogen Receptor 1 (*ESR1*) mRNA and enhanced ESR1 expression, while increased ESR1 further promoted *Mettl3* transcription. ESR1 inhibited the transcription of adipogenic factor *PPARγ*, ameliorating adipogenesis in Fibro/adipogenic progenitors (FAPs) [77]. In a similar context, studies have revealed that FTO levels are negatively correlated with m^6^A modifications during adipogenesis. Recent research has confirmed that FTO directly targets ATG5 and ATG7, regulating their expression in an m^6^A-dependent manner. In the absence of FTO, mRNAs of *ATG5* and *ATG7* accumulate higher levels of m^6^A, which are recognized by YTHDF2, leading to reduced degradation and consequently alleviated autophagy and adipogenesis [78]. FTO could regulate Catenin β-1 (CTNNB1) expression in a demethylating manner to promote lipogenesis [79]. Nicotinamide adenine dinucleotide phosphate (NADP) regulated mRNA m^6^A via FTO in vivo, and deletion of FTO blocked NADP-enhanced adipogenesis in 3T3-L1 preadipocytes [80]. ALKBH5 mediates RNA stability of *LCAT* through demethylation and affects chicken adipogenesis [81]. Furthermore, m^6^A modification has been shown to upregulate mitochondrial carrier 2 (MTCH2) expression, promoting adipogenesis in pig muscle preadipocytes. The reader protein YTHDF1 enhances the translation of MTCH2 by recognizing its m^6^A modification [82]. HNRNPC is positively associated with reduced adipogenesis during aging in an m^6^A-dependent manner [83]. The adipoq gene in the PPAR signaling pathway promotes adipogenesis in an m^6^A-YTHDF1-dependent manner [84]. Collectively, these findings contribute to our understanding of the molecular mechanisms underlying adipogenesis and open avenues for the development of novel therapeutic strategies for obesity (Figure 2).

### 3.2. M^6^A Regulates Lipid Metabolism

In recent years, numerous studies have highlighted the role of m^6^A in regulating lipid metabolism and expansion. In 2017, it was first proposed that the m^6^A modification is involved in lipid metabolism regulation in yeast cells [85]. Since then, extensive research has focused on elucidating the role of m^6^A in lipid metabolism within mammalian cells.

Transcriptomic analyses of m^6^A in adipose tissue have demonstrated that methylation processes in pigs are primarily involved in lipid metabolism and adipocyte differentiation, suggesting that m^6^A may serve as a key regulator of lipid metabolism. Notably, the proportion of m^6^A in the WAT of lean pigs was significantly higher than that in obese pigs, with lean pigs exhibiting lower expression of FTO and higher expression of METTL3 [86]. A subsequent study indicated that FTO mediates m^6^A modification to regulate triglyceride deposition in HepG2 cells, suggesting that FTO promotes lipid metabolism [87]. Further research showed that hepatocyte-specific knockdown of METTL3 inhibited fatty acid metabolism by reducing the mRNA methylation levels of fatty acid synthase [88]. Similarly, the knockdown of METTL3 or YTHDF2 in HepG2 cells inhibited lipid accumulation [89]. Additional studies have demonstrated that natural compounds and external stressors may influence lipid metabolism by modulating m^6^A modification [8,90]. m^6^A modification stabilizes the mRNA of the lipid-metabolizing enzyme ELOVL6 via the m^6^A reader IGF2BP3, leading to a rewiring of fatty acid metabolism with a reduction in palmitic acid accumulation [91]. Taken together, these findings underscore the involvement of m^6^A in the regulation of lipid metabolism.

### 3.3. M^6^A Regulates White Adipose Tissue Beiging

Traditionally, adipose tissue has been categorized into white adipose tissue (WAT) and brown adipose tissue (BAT), with WAT primarily serving as an energy reservoir through triglyceride storage, and BAT generating heat in response to various stimuli. However, this binary model has been expanded by recent findings showing that certain white adipocytes can undergo a transformation into brown-like adipocytes, termed beige fat [92,93]. This discovery has opened new avenues for research into the prevention and treatment of obesity [94].

The beiging of white adipose tissue is a crucial aspect of fat metabolism, with significant implications for obesity treatment [93,94]. In our study, we reported that METTL3 is induced during WAT beiging in mice by preventing thermogenic mRNAs, including Krüppel-like factor 9 (*Klf9*), in an m^6^A-dependent manner [95]. Similarly, another study has shown that METTL3 regulates beige adipocyte glycolysis, which impacts beige fat thermogenesis and beige preadipocyte proliferation [96]. Previous studies have highlighted the regulatory role of FTO in the beiging of white fat and energy metabolism [97,98]. Entacapone, an active FTO inhibitor, upregulates the m^6^A modification of forkhead box O1 *(FOXO1)* mRNA, promoting beige fat formation and thermogenesis in inguinal adipose tissue. Notably, when the m^6^A site of *FOXO1* is mutated, the beneficial effects of Entacapone in reducing high-fat diet-induced weight gain and fasting blood glucose levels in mice are blocked [99]. Similarly, deletion of FTO in vitro promotes thermogenesis and white-to-beige adipocyte transition. Mechanistically, FTO deficiency increases the m^6^A level of Hypoxia-inducible factor 1-α (*Hif1a*) mRNA, which is recognized by the m^6^A-binding protein YTHDC2, facilitating mRNA translation and increasing HIF1A protein abundance [100]. HIF1A then promotes the expression of thermogenic genes, including uncoupling protein 1 (UCP1), thereby facilitating the beiging of white adipose tissue. Additionally, we found that YTHDF1 facilitates the translation of bone morphogenetic protein 8b (*Bmp8b*) in an m^6^A-dependent manner to induce the beiging process [101] (Table 1). The recent study showed that upregulated m^6^A modification after acute exercise induces the formation of glycolytic beige fat in WAT [102]. In summary, these studies highlight the comprehensive role of m^6^A in beige fat biology and systemic energy homeostasis. However, the initiation of beige adipose tissue is unclear. In human cells and tissues, additional research is needed to clarify the role of m^6^A and provide a solid foundation for future therapeutic strategies aimed at obesity.

## 4. The Role of m^6^A in Glucose Metabolism-Related Diseases

Glucose metabolism serves as a vital energy source for processes such as aerobic oxidation, anaerobic fermentation, and the pentose phosphate pathway. Type 2 diabetes (T2D), a multifaceted metabolic disorder, is characterized by hyperglycemia and dyslipidemia. Recent studies highlight the pivotal role of m^6^A methylation modification in the pathogenesis of T2D. Evidence suggests that glucose levels dynamically regulate m^6^A in T2D patients, with high glucose conditions suppressing FTO expression while enhancing the levels of the methyltransferase complex, including METTL3, METTL14, and WTAP. Key regulators of glucose homeostasis, such as FOXO1 and glucose-6-phosphatase catalytic subunit 1 (G6PC), as well as diacylglycerol O-acyltransferase 2 (DGAT2)—an enzyme essential for triglyceride synthesis and lipid storage—are implicated in this regulatory axis. Overexpression of FTO has been shown to upregulate *FOXO1, G6PC*, and *DGAT2* mRNA, thereby disrupting glucose and lipid metabolism [103,104].

Regulation of insulin secretion represents another crucial aspect of maintaining glucose homeostasis. METTL3 has been identified as a suppressor of hepatic insulin sensitivity via m^6^A modification of fatty acid synthase (*FASN*) mRNA, thereby promoting fatty acid metabolism [88]. Moreover, in human adipose tissue, *FASN* and *GCK* are potential biomarkers of insulin resistance and may be involved in the development of T2D via their m^6^A modification [105]. Hepatocyte-specific knockdown of METTL3 in mice alleviated HFD-induced metabolic disorders by slowing weight gain, reducing lipid accumulation, and improving insulin sensitivity [106,107]. Conversely, the loss of *Mettl3* results in pancreatic β-cell failure and hyperglycemia. METTL4, another m^6^A methyltransferase, has been shown to induce β-cell death and impair β-cell differentiation when acutely depleted in adult mice, leading to reduced insulin secretion and glucose intolerance [108]. In humans and mice with insulin resistance, METTL14 expression differs significantly between BAT and WAT in the context of its correlation with insulin sensitivity [109]. Targeting METTL3/14 in vitro increases the protein levels of ACLY and SCD1, as well as triglyceride and cholesterol production and accumulation of lipid droplets [110]. Consistently, WTAP plays a key role in maintaining β-cell function by regulating m^6^A mRNA modification depending on METTL3, and the downregulation of WTAP leads to β-cell failure and diabetes [111]. Downregulation of YTHDC1 leads to islet β-cell failure and diabetes [112]. Overexpression of YTHDC2 in the livers of obese mice improved liver steatosis and insulin resistance by decreasing the mRNA stability of lipogenic genes and inhibiting gene expression [113].

MASLD, the most common chronic liver disease, is a common complication of type 2 diabetes mellitus (T2DM). Hepatic lipid deposition is a key factor in the development of MASLD. The methyltransferase METTL3 inducing the upregulation of RUBICON is involved in impaired autophagic flux and lipid metabolism in an m^6^A-YTHDF1-dependent manner in NAFLD [114]. Modulation of the Mettl3–m^6^A–YTHDF1 axis has the potential to improve mitochondrial function, alleviate MASLD symptoms, and decrease the likelihood of disease progression [115]. METTL14/m^6^A-based epitranscriptomic reprogramming impairs adipose ADRB signaling and lipolysis, promoting obesity, MASLD, and metabolic disease [116,117]. Silencing METTL14 reduced weight gain and mitigated adverse liver function indices, inflammation, hepatic steatosis, and structural damage in NAFLD mice [118]. FTO transactivation and m^6^A demethylation on mRNA of lipogenic genes induced lipogenic gene activation and lipid accumulation during NAFLD and were mediated by glucocorticoid receptor [119]. Hepatocyte-specific deletion of *Alkbh5* improves glucose tolerance and mitigates metabolic dysfunction-associated fatty liver disease (MAFLD) in obesity by inhibiting GCGR–cyclic adenosine monophosphate (cAMP) and EGFR–PI3K–AKT–mTORC1 signaling. Targeted knockdown of *Alkbh5* reverses T2DM and MAFLD in diabetic mice [120]. ALKBH5 alleviates hepatic lipid deposition and impaired autophagic flux by removing the m^6^A modification on *VPS11* mRNA to promote its translation [121]. Decreased ALKBH5 causes increased m^6^A modification and increased expression of ATG12 in a demethylase activity-dependent manner, thereby promoting autophagy and preventing hepatic steatosis [122]. YTHDF2-mediated translation of SLC9A6-126aa is largely responsible for the detrimental effects of circ-SLC9A6 on hepatic lipid metabolism [123]. Collectively, these findings underscore the critical role of m^6^A in glucose metabolism and its potential as a therapeutic target for T2D and related metabolic diseases (Table 2).

## 5. The Role of m^6^A in Glucose and Lipid Metabolism in Cancer Cells

Metabolic reprogramming is a hallmark of cancer pathogenesis, characterized by abnormal regulation of metabolic pathways [124]. Among these, aerobic glycolysis (the Warburg effect) and lipid metabolism disorders are two prominent pathways associated with cancer cell survival and proliferation [125,126]. Emerging evidence suggests that m^6^A methylation, a dynamic and reversible RNA modification, plays a pivotal role in regulating cancer metabolic reprogramming. By modulating the stability, translation efficiency, and splicing of key metabolic genes, m^6^A influences the metabolic adaptations that enable cancer cells to thrive under adverse conditions. These findings provide new insights into the molecular mechanisms underpinning cancer metabolism and offer potential therapeutic targets.

### 5.1. M^6^A Regulates Glycolysis in Cancer Cells

The Warburg effect, characterized by the activation of glycolysis and increased lactic acid fermentation, is a hallmark of cancer cell metabolism [127]. Recent studies have uncovered that m^6^A methylation plays a crucial role in regulating key enzymes and pathways involved in this metabolic shift. In patients with colorectal cancer (CRC), METTL3 enhances the stability of hexokinase 2 (*HK2*) and glucose transporter *GLUT1* mRNAs by interacting with their 3′UTR regions, with the stability of *HK2* and *GLUT1* being mediated by IGF2BP2 and IGF2BP3, respectively. This stabilization ultimately activates the glycolytic pathway [128]. Meanwhile, another study found that METTL3 enhances the HK2 stability through YTHDF1-mediated m^6^A modification, thereby promoting the Warburg effect of human cervical cancer (CC) [129]. Circ-CTNNB1 interacts with RBM15 and subsequently promotes the expression of HK2 through m^6^A modification to facilitate the glycolysis process and activate osteosarcoma progression [130]. Similarly, WTAP has been shown to bind to the m^6^A sites within the 3′UTR of *HK2*, further enhancing its mRNA stability. Functional studies reveal that WTAP promotes cell proliferation and nuclear glycolysis, while its loss inhibits tumor growth [131]. Down-regulated FTO and ALKBH5 co-operatively activate FOXO signaling through m^6^A methylation modification in *HK2* mRNA mediated by IGF2BP2 to enhance glycolysis in colorectal cancer [132] (Table 3).

Additional evidence links m^6^A modification to other cancer-related pathways. In clear cell renal cell carcinoma (ccRCC), METTL3-m^6^A modification reduces dihydrolipoamide branched chain transacylase E2 (DBT) expression and promotes tumor progression and corrects the lipid metabolism disorder [133]. In bladder cancer (BCa), m^6^A modification mediated by METTL14 promotes the expression of lncDBET and then activates the PPAR signaling pathway to promote the lipid metabolism of cancer cells, thus promoting the malignant progression of BCa in vitro and in vivo [134]. METTL3 regulates the AFF4/NF-kB/MYC signaling axis, promoting BCa progression [135]. Casein kinase 2 (CK2), particularly its catalytic subunit CK2α, plays a critical role in glycolysis. ALKBH5, by specifically recognizing the m^6^A site within the 3′UTR of *CK2α* mRNA, reduces its stability, thereby inhibiting cell glycolysis and proliferation in BCa [136]. The YTHDF1/eEF-2 complex and IGF2BP3 interact with the m^6^A-modified 5′UTR of pyruvate dehydrogenase kinase 4 (*PDK4*), promoting its translation, elongation, and mRNA stability, thus increasing the expression of PDK4 and glycolysis of cancer cells [137]. Collectively, these findings demonstrate that m^6^A modification regulates cancer progression by modulating glycolytic enzymes and related signaling pathways, thereby influencing glucose metabolism.

### 5.2. M^6^A Affects Cancer Cell Lipogenesis

Lipid metabolism reprogramming is a hallmark of cancer cells, characterized by the upregulation of several lipogenic enzymes [138]. METTL3 enhances the stability of the long non-coding RNA (lncRNA) LINC00958, which promotes lipogenesis in hepatocellular carcinoma (HCC). LINC00958 modulates the expression of key lipogenesis-related factors, including sterol regulatory element-binding protein 1 (SREBP1), FASN, stearoyl-CoA desaturase, and acetyl-CoA carboxylase 1 (ACC1) [139]. RBM15 mediates the activation of ACLY by regulating m^6^A modification in an IGF2BP2-dependent manner, thereby driving lipogenesis and exacerbating the malignant characteristics in gastric cancer [140]. Furthermore, overexpression of FTO in the liver promotes triglyceride accumulation by demethylating the m^6^A sites of lipogenic genes and stabilizing the genes [141]. FTO promoted the formation of lipid droplets in esophageal cancer cells by enhancing HSD17B11 expression [142]. In pancreatic neuroendocrine neoplasms, ALKBH5 over-expression was found to increase the expression of Fatty acid-binding protein 5 (FABP5) in an m^6^A-IGF2BP2 dependent manner, leading to disorders in lipid metabolism [143]. IGF2BP3 regulated *SCD* mRNA m^6^A modifications via IGF2BP3–METTL14 complex, thereby enhancing cervical cancer proliferation, metastasis, and lipid metabolism [144]. METTL16 restrains papillary thyroid carcinoma progression through SCD1-activated lipid metabolism in cooperation with YTHDC2 [145]. METTL3 and YTHDF1 regulate lipid metabolism via the autophagy pathway in NAFLD [114]. In cervical cancer, the upregulation of LRP6 through YTHDF3-mediated m^6^A modification results in increased expression of FASN and ACC1, leading to both lipolysis of lipid droplets and synthesis of free fatty acids [146]. Histone lactylation in HCC induces increased expression of YTHDC1, increasing the stability of m^6^A-modified *NEAT1*, thus facilitating HCC progression via hepatocellular lipid metabolism remodeling [147] (Table 4). These findings highlight the role of m^6^A methylation in modulating lipogenesis in cancer cells, suggesting its potential as a therapeutic target for lipid metabolism-associated malignancies.

## 6. Clinical Research on m^6^A Targeted Therapy Needs Further Investigation

As multiple studies have elucidated the role of m^6^A in obesity and related metabolic diseases, drug development and clinical research targeting m^6^A have become a prominent trend. To date, there are no m^6^A regulators available for clinical practice. Since the discovery of FTO as an m^6^A demethylase, it has become a focal point for m^6^A-targeted therapy. FTO directly binds to m^6^A substrates within cells [148]. Following this, selective FTO inhibitors were developed, which can specifically inhibit FTO activity and thus upregulate m^6^A levels in cells [149,150,151,152]. Additionally, entacapone, a catechol-O-methyltransferase inhibitor used in Parkinson’s disease, was also identified as an FTO inhibitor, influencing metabolic homeostasis by selectively inhibiting FTO activity [99]. R-2-hydroxyglutarate (R-2HG) abrogates FTO/m^6^A/YTHDF2-mediated post-transcriptional upregulation of phosphofructokinase platelet (PFKP) and lactate dehydrogenase B (LDHB) (two critical glycolytic genes) expression and thereby suppresses aerobic glycolysis [153]. Meclofenamic acid (MA), as an FTO demethylation inhibitor, downregulated advanced glycation end product (AGE)-treated podocytes, subsequently decreasing podocyte fatty acid accumulation [154]. Recently, researchers have designed MA analogs FB23-2 and Dac51, which exhibit significantly improved activities compared with MA. FB23-2 promotes the differentiation/apoptosis of human acute myeloid leukemia (AML) cells and inhibits the progression of primary cells in xenotransplanted mice. Dac51 treatment impairs the glycolytic activity of tumor cells and restores the function of CD8^+^ T cells, thereby inhibiting the growth of solid tumors in vivo [155]. Beyond FTO, other m^6^A regulatory proteins also present promising therapeutic targets for metabolic diseases. METTL3 activators may inhibit BMSC adipogenesis and differentiation, offering an effective obesity treatment [69]. Activation of the METTL3 complex by its chemical ligand methyl piperidine-3-carboxylate (MP3C) could promote beige and brown adipose tissue thermogenesis, improving systemic metabolism to alleviate obesity [95]. Treatment of tumors with STM2457, a highly potent and selective first-in-class catalytic inhibitor of METTL3, leads to reduced AML growth and an increase in differentiation and apoptosis [156]. Treating the fathers with STM2457 restores obesity-reduced sperm count and decreases Wt1 N6-methyladenosine level in the mouse testes of the offspring [157]. Additionally, preclinical studies of STC-15 show promise in inhibiting tumor growth via direct anti-tumor effects and anti-cancer immune responses [158]. Structure-based virtual screening of FDA-approved drugs identified tegaserod as a potential YTHDF1 inhibitor. Tegaserod blocked the direct binding of YTHDF1 with m^6^A-modified mRNAs and reduced the viability of patient-derived AML cells in vitro and prolonged survival in patient-derived xenograft models [159]. Recently, studies showed that natural products from traditional medicine could be used as a chemical library for m^6^A-targeting anticancer drug discovery [160]. Collectively, m^6^A plays a crucial role in obesity and various other diseases. However, clinical research targeting m^6^A requires further investigation to better understand its potential therapeutic applications.

## 7. Conclusions and Future Directions

As multiple studies of obesity, a critical global health challenge, are closely linked to various metabolic disorders and an increased risk of mortality. Research has demonstrated that obesity induction consistently leads to a decline in adipose tissue [161], a process that may be dynamically influenced by epigenetic regulation. Among these regulatory mechanisms, m^6^A methylation—a reversible and dynamic post-transcriptional modification—plays a pivotal role in lipogenesis, lipid metabolism, and insulin sensitivity by modulating gene expression networks in an m^6^A-dependent manner. As mentioned above, although the “writers”, ”erasers”, and “readers” of m^6^A have distinct roles in biological processes, they always cooperate to maintain the body’s homeostasis [35,38,129,162]. Furthermore, in human cohorts, the study found that the writers WTAP and VIRMA, the eraser ALKBH5, and reader proteins such as YTHDF1, YTHDF2, and YTHDC1 are associated with obesity by comparing gene expression of m^6^A regulators in adipose tissue between individuals with obesity and lean controls. They also observed significant correlations with clinical parameters for VIRMA, WTAP, and ALKBH5 [163].

However, further research on m^6^A methylation in obesity and its related metabolic disorders, such as T2D and MASLD, is still needed. Several key challenges persist: (1) The origin and plasticity maintenance mechanisms of beige adipocytes have yet to be fully elucidated; (2) a comprehensive analysis of the complex m^6^A modification network in inter-organ metabolic communication is unclear; and (3) the development of m^6^A-targeted interventions faces significant specificity and safety challenges. Therefore, further research is essential to elucidate the mechanisms and functions of m^6^A methylation in these metabolic diseases, thereby laying the groundwork for the development of m^6^A-targeted therapies and novel treatment strategies.

## Figures and Tables

**Table 1:** m^6^A regulates white adipose tissue beiging.

m^6^A Regulators	m^6^A “Readers”	Key Mechanism	References
METTL3	-	↓ the thermogenic mRNAs (including *Klf9*) degradation	[95]
	IGF2BP2	↑ mRNA stability of key glycolytic genes in beige adipocytes	[96]
FTO	-	Inhibitor of FTO → ↑ the m^6^A of *FOXO1* → ↑ white adipose tissue beiging	[99]
	YTHDC2	FTO deficiency → ↑ the m^6^A of *Hif1a* → YTHDC2 recognized → ↑ HIF1A protein → ↑ white adipose tissue beiging	[100]
-	YTHDF1	Recognized the m^6^A of *Bmp8b* → ↑ white adipose tissue beiging	[101]

“-”, none.

**Table 2:** The role of m^6^A in glucose metabolism-related diseases.

m^6^A Regulators	m^6^A “Readers”	Mechanism	Cancer	Reference
METTL3	-	↑ mRNA level of *FASN* → ↑ fatty acid metabolism	Insulin sensitivity	[88]
METTL3/14	-	↑ protein level of ACLY and SCD1 → ↑ lipid droplets	NAFLD	[110]
METTL3	YTHDF1	↑ RUBICON → ↓ lipid metabolism	NAFLD	[114]
METTL14	-	↓ ADRB signaling and lipolysis → ↑ obesity and MASLD	MASLD	[116,117]
FTO	-	↑ lipogenic gene → ↑ lipid accumulation	NAFLD	[119]
ALKBH5	-	↓ cAMP and EGFR-PI3K-AKT-mTORC1 signaling → ↑ glucose tolerance	MAFLD	[120]
		↑ the translation of VPS11 → ↓ lipid deposition	NAFLD	[121]
-	YTHDC2	↓ mRNA stability of lipogenic genes → ↑ liver steatosis	Insulin resistance and NAFLD	[113]

“-”, none.

**Table 3:** M^6^A regulates glycolysis in cancer cells.

m^6^A Regulators	m^6^A “Readers”	Mechanism	Cancer	Reference
METTL3	IGF2BP2/3	↑ mRNA stability of *GLUT1* → ↑ glycolysis pathway	Colorectal cancer	[128]
	IGF2BP2	↑ mRNA stability of *HK2* → ↑ glycolysis pathway		
	YTHDF1	↑ mRNA stability of *HK2* → ↑ Warburg effect	Cervical cancer	[129]
RBM15	-	RBM15 interacted with Circ-CTNNB1 → ↑ HK2 expression→ ↑glycolysis	Osteosarcoma	[130]
WTAP	YTHDF1	↑ mRNA stability of *HK2* → Recognized by YTHDF1 → ↑ HK2 protein → ↑ Warburg effect	Gastric cancer	[131]
FTO and ALKBH5	IGF2BP2	↑ FOXO signaling → ↑ HK2 recognized by IGF2BP2 → ↑ glycolysis	Colorectal cancer	[132]

“-”, none.

**Table 4:** M^6^A affects cancer cell lipogenesis.

m^6^A Regulators	m^6^A “Readers”	Mechanism	Cancer	Reference
METTL3	-	↑ mRNA stability of *LINC00958* → ↑ lipogenesis	Hepatocellular carcinoma	[139]
METTL3	YTHDF1	Regulating lipid metabolism via the autophagy pathway	Nonalcoholic fatty liver disease	[114]
METTL14	IGF2BP3	↑ *SCD* → lipid metabolism	Cervical cancer	[144]
RBM15	IGF2BP2	↑ *ACLY* → recognized by IGF2BP2 → ↑ lipogenesis	Gastric cancer	[140]
FTO	-	↑ mRNA stability of lipogenic genes		[141]
	-	↑ HSD17B11→ ↑ lipogenesis	Esophageal cancer	[142]
ALKBH5	IGF2BP2	↑ FABP5 expression → disorder the lipid metabolism	Pancreatic neuroendocrine neoplasms	[143]
-	YTHDF3	↑ LRP6 expression → ↑ FASN and ACC1 expression → lipid metabolism	Cervical cancer	[146]
-	YTHDC1	↑ mRNA stability of *NEAT1* → lipid metabolism	Hepatocellular carcinoma	[147]
-	YTHDC2	SCD1-activated lipid metabolism	Thyroid cancer	[145]

“-”, none.

## Abbreviations

The following abbreviations are used in this manuscript:

ALKBH5	AlkB homolog H5
ATG	Autophagy protein
BAT	Brown adipose tissue
C/EBP	CCAAT/enhancer-binding protein
EIF3	Eukaryotic initiation factor 3
FTO	Fat mass and obesity associated
HIF1A	Hypoxia-inducible factor 1-α
HNRNP	Heterogeneous nuclear ribonucleoproteins
IGF2BP	Insulin-like growth factor 2 mRNA-binding protein
iWAT	Inguinal white adipose tissue
METTL	Methyltransferase like
MP3C	Methyl piperidine-3-carboxylate
mRNA	Messenger RNA
m6A	N6-methyladenosine
m6A-IP	m6A-specific immunoprecipitation
MSCs	Mesenchymal stem cells
MASLD	Metabolic dysfunction-associated steatotic liver disease
NAFLD	Non-alcoholic fatty liver disease
RBM15	RNA binding motif protein 15
UCP1	Uncoupling protein 1
UTR	Untranslated region
VIRMA	Vir like M6A methyltransferase associated
WAT	White adipose tissue
WTAP	WT1-associated protein
YTHDC	YT521-B homology domain containing
YTHDF	YT521-B homology domain family
ZC3H13	Zinc finger CCCH-type containing 13
